# Optimization and application of loop‐mediated isothermal amplification technique for sex identification in red‐whiskered bulbul (*Pycnonotus jocosus*)

**DOI:** 10.1002/ece3.9401

**Published:** 2022-10-05

**Authors:** Phanupong Changtor, Yash Munnalal Gupta, Nonglak Yimtragool

**Affiliations:** ^1^ Department of Biology, Faculty of Science Naresuan University Phitsanulok Thailand

**Keywords:** colorimetric assay, LAMP, red‐whiskered bulbul, sex identification

## Abstract

The red‐whiskered bulbul (*Pycnonotus jocosu*s) is a popular avian species in Thailand and many other countries. The red‐whiskered bulbul has a high economic value, but breeding is challenging since sex identification is difficult. The PCR method is now used for sex identification. However, PCR amplification and post‐PCR analysis necessitate the use of a laboratory equipped with specialized scientific instruments, which is inconvenient for field operations. This research describes a method for amplification of DNA samples using the loop‐mediated isothermal amplification (LAMP) approach, which is a molecular biology methodology for isothermal amplification that is extremely sensitive, fast, and easy for post‐LAMP product visualization. Herein, total of 23 blood samples were collected and DNA was extracted. Two sets of LAMP primers were designed for *CHD‐Z* and *CHD‐W* genes. The colorimetric assay was used to investigate the best conditions for LAMP reactions and post‐LAMP product visualization. LAMP reactions for sex identification were compared to traditional PCR in terms of sensitivity and specificity. LAMP reactions were found to be 10‐fold more sensitive than PCR at 1 ng of DNA. When compared to electrophoresis analysis, the visualization with colorimetric assay using GelRed® and SYTO™ 9 was 100% accurate. The optimal LAMP condition tested simple DNA extracted from bird feathers using the HotSHOT technique. The result showed that the optimal condition could distinguish the sex of red‐whiskered bulbuls totally and accurately. A powerful method for red‐whiskered bulbul sex identification is demonstrated in this study, which can be used in field studies because it is quick and easy to perform, has high sensitivity, and does not require advanced scientific equipment.

## Graphical abstract

This research describes a method for DNA amplification using the LAMP technique, which is an isothermal amplification that is demonstrated a visual, rapid, and yet specificity but higher than the gold standard laboratory‐based method. In addition, we presented the limits of the LAMP technique (sensitivity and specificity) for sex identification of the Red‐whiskered bulbul and the colorimetric assays method for application in field studies.

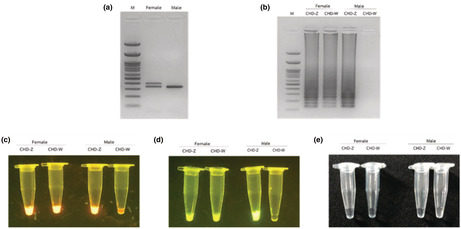

## 1 INTRODUCTION

Red‐whiskered bulbul (*Pycnonotus jocosus*) is a passerine bird native to South‐east Asia which is a popular in Thailand, Malaysia, Myanmar, and Singapore (Linnebjerg et al., 2010; Sunthorn, 2003), as well as some parts of the United States (Florida, Hawaii, Los Angeles; Rand, 1980; Van Riper et al., 1979) and Australia (Sydney, Melbourne, Adelaide; Mo, 2015). However, after carefully examining the literature, it appears that this species has been released or escaped from captivity and is now being regarded as an invasive species in some areas, including Spain (Valencia; Domínguez‐Pérez & Gil‐Delgado, 2022). However, based on observations made in Thailand, it appears that the red‐whiskered bulbul population has declined as a result of the expansion of the agricultural sector, extensive plantations and the fragmentation and reduction of the bird's habitat. In Thailand, red‐whiskered bulbul was carefully raised in birdcage because the bulbul is associated with human tradition, voice competition, and as a popular trend for being a pet to listen to a beautiful voice (Techachoochert & Round, 2013). The economic value of the red‐whiskered bulbul is extremely high due to its widespread popularity. The birds can cost as much as 1500 USD in Thailand. However, the red‐whiskered bulbul is a monomorphic bird species with no phenotypic differences between males and females. Sex identification of a bird is essential for identifying its gender before trading. It is crucial to note that if sex identification is erroneous, the study on population structure may be incorrectly estimated. The precise estimates of vitality rates and the sex ratio are essential for effective conservation and management measures such as deer (Clutton‐Brock et al., 2002), bear (Jerina & Adamič, 2008), and bird (Cerit & Avanus, 2007). In the past, sex identification was based on behavior observation and studies of their external appearance, as well as examination of the pelvis and the cloaca (Hildebrandt et al., 1995), measurement of steroid levels (Hirschenhauser et al., 1999) and chromosome karyotype (Manee, 2004). The techniques mentioned above have numerous issues, are complicated, and necessitate specialized knowledge. The ornithologist or bird breeders could be mistaken in sex identification.

The polymerase chain reaction (PCR) was developed in the 1980s and has been shown to be more accurate in sex identification. This technique is especially useful for amplifying the duplicated Chromo‐Helicase‐DNA‐binding protein (*CHD*) sex‐chromosome‐specific gene, which is located on the Z and W chromosomes (Griffiths et al., 1998; Yimtragool & Changtor, 2022). Females in birds have heterogametic sex (ZW), whereas males have homogametic sex (ZZ). Sex determination in birds is possible due to evolutionary differences in the intronic region of *CHD‐Z* and *CHD‐W*. Several molecular techniques based on PCR and the *CHD* sex‐specific gene, such as restriction fragment length polymorphism (Bermúdez‐Humarán et al., 2002), random amplified polymorphic DNA (Lessells & Mateman, 1998), amplified fragment length polymorphism (Griffiths & Orr, 1999), capillary electrophoresis (Lee et al., 2010), and real‐time PCR (Brubaker et al., 2011; Chang et al., 2008; Morinha et al., 2013), have been used to determine the sex of the bird. These techniques required expensive equipment and no matter how precise.

These issues have been addressed by the loop‐mediated isothermal amplification technique (LAMP), a new, simple, rapid, high specificity, and efficient gene amplification method developed by Notomi et al. (2000). The LAMP technique is advantageous because it uses isothermal temperature; *Bst* polymerase has strand displacement activity; and four primers recognize six regions (Notomi et al., 2000). After combining lateral flow dipstick (Moonga et al., 2020; Sharma et al., 2021), chemical (Papadakis et al., 2022; Poole et al., 2017), and fluorescence dyes addition (Wong et al., 2017), visualization products can be observed directly with the naked eye, eliminating the need for gel electrophoresis. As a result, LAMP can be used in a field study because the reaction can be run in the field. LAMP has been used in many field studies as a molecular technique, such as the detection of malaria parasites (*Plasmodium falciparum*; Paris et al., 2007), the detection of *Fusarium graminearum*, the cause of Downy Mildew in wheat (*Triticum aestivum* L.), and also for the identification of human DNA (Kanchanaphum, 2018). Moreover, LAMP has been used to determine the sex of various plants, including papaya (Tsai et al., 2016), asparagus (Shiobara et al., 2011), and spinach (Fujita et al., 2017). In raptor species, sex identification based on LAMP also has been developed (Centeno‐Cuadros et al., 2016). The LAMP method has been shown to distinguish between male and female birds. In the visualized product of LAMP for sex identification, SYBR green I and ethidium bromide staining have been employed. However, there are a variety of colorimetric assays for visualizing LAMP products, and the most commonly reported disadvantage of LAMP is its high rate of false positives (Hardinge & Murray, 2019). Each method has its own set of benefits and drawbacks, such as UV exposure or sensitivity for DNA detection in field studies. Therefore, more research into the applicability of LAMP in the field appears to be required.

In this study, we developed a test kit for sex identification of the red‐whiskered bulbul using the LAMP technique. Two primer sets to amplify the sex‐specific genes *CHD‐Z* and *CHD‐W* were designed in each tube and investigated the optimal conditions for LAMP reactions. The sensitivity and specificity of colorimetric assays by GelRed®, SYTO™ 9, and CuSO_4_ were also examined and compared to the agarose gel electrophoresis method. The high‐efficacy colorimetric visualization of LAMP detection will be extremely useful in the bird breeding industry for quickly determining the sex of red‐whiskered bulbul.

## 2 MATERIALS AND METHODS

### 2.1 Sample collection

**FIGURE 1:**
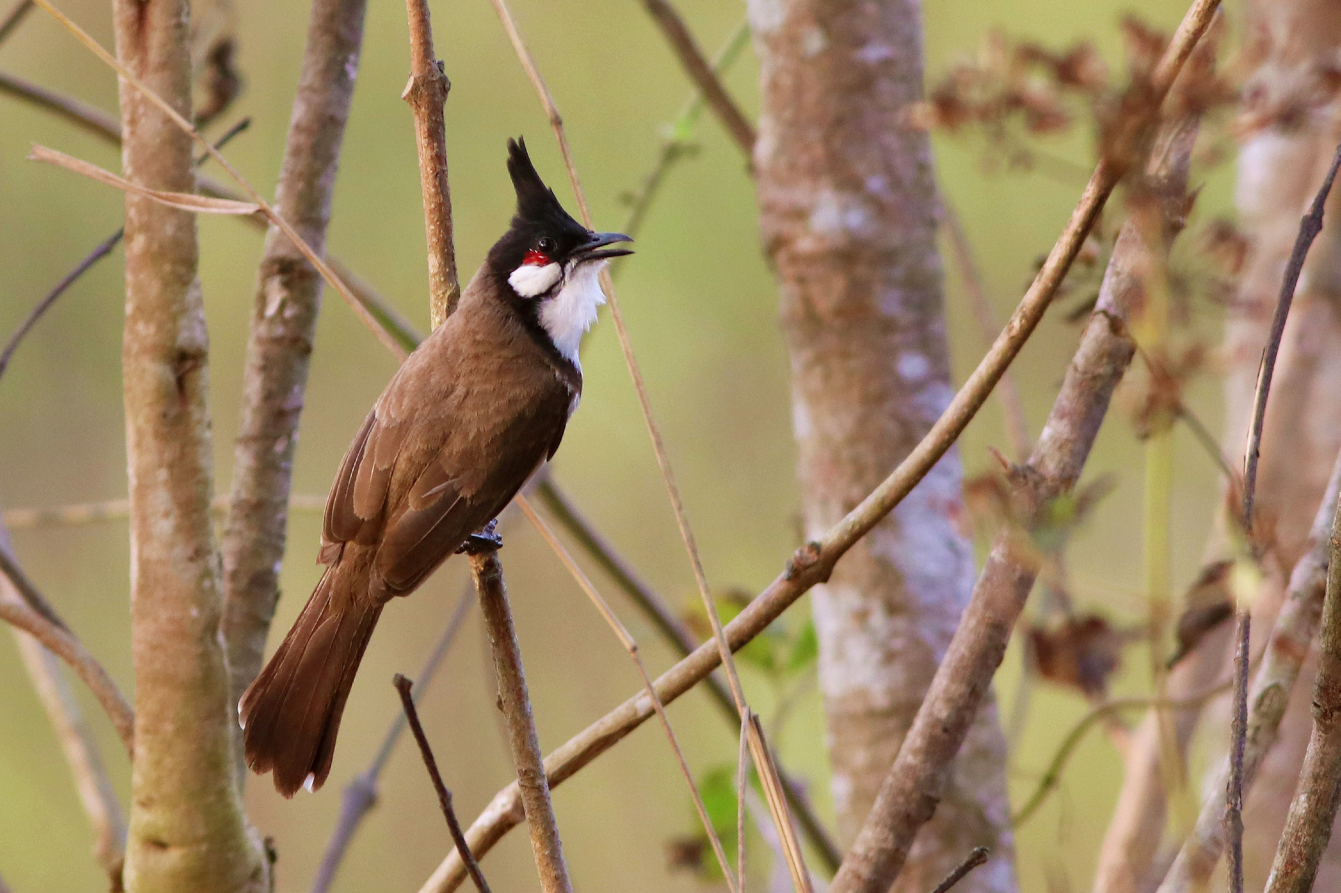
Red‐whiskered bulbuls (*Pycnonotus jocosus*) at Khao Yai National Park, Nakhon Ratchasima, Thailand, in a winter season.

The red‐whiskered bulbuls (*Pycnonotus jocosus*; Figure 1) were obtained from a local farm in Thailand. Blood samples were collected from the toenail by cutting with nail clippers and dropped on paper around 10 μl. After nail cutting, Ideal® Animal Health Blood Stop Powder (Neogen Animal Safety, USA) was used to stop the bleeding (Sakas, 2002). Each sample was kept in a plastic bag and stored at −20°C until used. Animal experiment protocols were approved by the Naresuan University Animal Ethics Committee: registration number NU‐AE620512.

### 2.2 DNA extraction using lysis buffer

DNA was extracted from blood using a modified lysis buffer composition adapted from Bayard de Volo et al. (2008). The 10‐μl blood sample absorbed paper was soaked in a microcentrifuge tube containing 300 μl of 1× TNE (Tris 50 mM, NaCl 100 mM and EDTA 25 mM), 30 μl of 1 M Tris–HCl, 22.5 μl of 25 mg/ml proteinase K, 5 μl of 25% SDS and 40 μl of 1 M DTT. The microcentrifuge tube containing the blood sample and chemicals was incubated at 55°C for 2 h. Protein was precipitated using 300 μl of 7.5 M ammonium acetate. The reaction tube was vortexed, frozen at −20°C for 30 min, and centrifuged at 15,000 *g* for 30 min at 4°C. The supernatant was transferred into a new tube. Isopropanol was added in an equal volume of supernatant, mixed by gentle flip‐flopping, and stored at −20°C overnight. The following day, it was centrifuged at 15,000 *g* for 30 min at 4°C, and the isopropanol was discarded. The tube containing precipitate was washed with 400 μl of 70% ethanol and centrifuged at 15,000 *g* for 2 min at 4°C. The remaining ethanol was then discarded. After drying at room temperature, the DNA pellet was dissolved in 20 μl of nuclease‐free water and stored at −20°C until needed.

### 2.3 Loop‐mediated isothermal amplification primer design

**FIGURE 2:**
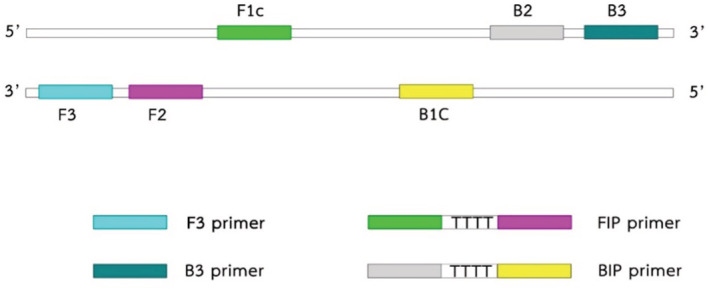
Schematic diagram of LAMP primer sets designed for *CHD‐Z* and *CHD*‐*W* genes. Six different regions were designed for four primers: two outer primers (F3 and B3) and two inner primers (FIP and BIP) were generated from the inner regions joined by the TTTT‐linker. The FIP primer pairs are F1c and F2 and the BIP primer pairs are B2 and B1c.

**FIGURE 3:**
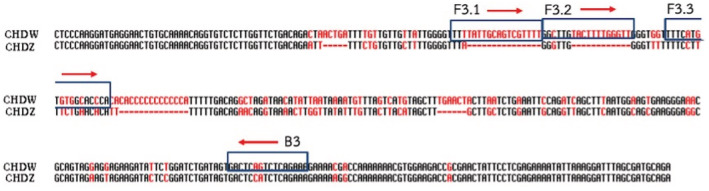
The location of the candidates of outer primers (F3.1‐F3.3/B3) along the alignment of *CHD‐Z* and *CHD‐W* gene. The red letter represent a nucleotide base, which is different between the *CHD‐Z* gene and the *CHD‐W* gene.

Two primer sets for *CHD‐Z* and *CHD‐W* sex‐specific genes were designed using the sequences derived from GenBank (www.ncbi.nlm.nih.gov.). First, the sequences of *CHD‐Z* (accession no. KM196532.1) and *CHD‐W* (accession no. KM196531.1) were aligned to find their differences. Then, PRIMER EXPLORER V5 software was used to obtain candidate primers. The primer sets were chosen based on optimal conditions, GC content, and sequence differences between *CHD‐Z* and *CHD‐W*. Each primer set consists of two pairs of forward and reward primers: outer primers (F3/B3) and internal primers (FIP/BIP). The internal primers for loop‐forming recognize four regions of F1c, F2, B1c, and B2. FIP is composed of F1c and F2 linked with four bases of TTTT, BIP is composed of B1c and B2 similarly linked with TTTT (Figure 2). Three forward outer primers (F3.1, F3.2, and F3.3) were designed specifically for the *CHD‐W* gene to reduce the chances of DNA amplification crosslinking, resulting in false‐positive and amplified using PCR with an annealing temperature of 58°C (Figure 3).

### 2.4 Loop‐mediated isothermal amplification reaction for sex identification

The optimal LAMP reactions were tested using different amplification temperatures (ranged between 60 and 64°C) and outer/inner primer concentration (ranged between 1:2 and 1:8). Two sets of LAMP mixture for *CHD‐Z* and *CHD‐W* gene (Table 1) detection were prepared to consist of 8 U *Bst* DNA polymerase (NEW ENGLAND BioLabs®), 1× Isothermal amplification buffer, 6 mM MgSO_4_, 1.4 mM dNTPs, 0.8 μM FIP/BIP primers, 0.2 μM F3/B3 primers, and 1 μg template DNA. The total volume was adjusted to 25 μl using nuclease‐free water. Reactions were incubated for 80 min at 63°C using the heat box incubator. The presence of LAMP products were detected using 2% agarose gel electrophoresis and stained with ethidium bromide for visualization under UV conditions.

**TABLE 1:** LAMP primers for the amplification of *CHD‐W* and *CHD‐Z* genes

Gene	Primer	Sequence (5′–3′)
*CHD‐Z*	F3	TCTGACAGAATTTTTCTGTGTT
	B3	TCACTATCAGATCCGGAGTA
	FIP	CCAAGTTTTACCTGTTCTGTCAAAATTTT GCTTTTGGGGTTTAGGGT
	BIP	TTGTTACTTACATAGCTTGCTTGCTTTTTT ACTTCTACTGCGCCTCC
*CHD‐W*	F3	GGCTTGTACTTTTGGGTT
	B3	GTTTTCTTTCTGAGACTGAGTC
	FIP	AGTTCAAAGCTACATGACTAAACATTTT CCCCCCCATTTTTGACAGG
	BIP	ATTCCAGATCAGCTTTAATGGAAGTTTTT CAGATCCAGAATATCTTCTCCTC

### 2.5 Sensitivity of colorimetric assays

DNA sample concentrations were measured using spectrophotometry method with nanodrop device (Thermo Fisher). In ten‐fold serial dilutions, a DNA concentration of 10^3^ ng was diluted to 10^−6^ ng. Diluted DNAs were amplified by the optimized condition of LAMP reactions and stained with 1 μl of 1000× GelRed® (Biotium), 0.05 mM SYTO™ 9 (Thermofisher), and 100 mM CuSO_4_ (Sigma). Except for the CuSO_4_‐stained products, other amplified products were visualized under UV light. LAMP products were visualized using a colorimetric assay, and the results were compared using gel electrophoresis. The experiment was carried out in triplicate.

### 2.6 Specificity of colorimetric assays

Blood samples were collected from twenty‐three red‐whiskered bulbul specimens of unknown sex. DNA was extracted from the blood using the method described above. A volume of 1 μl DNA was amplified by LAMP. The LAMP reaction's results were confirmed using conventional PCR.

### 2.7 Sex determination by conventional PCR

The sex‐specific primers of the *CHD‐Z* gene and the *CHD‐W* gene, P2/P8 were applied for sex identification (Changtor & Yimtragool, 2020; Tana & Panprommin, 2014) The reactions were carried out using MyTaq™ HS Mix (Hot start PCR, Bioline; 2× buffer 5 μl, 10 μM P8 1 μl, 10 μM P2 1 μl, nuclease‐free water 2 μl, and DNA template 1 μl). The PCR reactions were conducted with thermal cycler (Bio rad). The preincubation condition was performed for one cycle of 95°C for 5 min, followed by 35 cycles of 95°C for 45 s, 58°C for 45 s, and 72°C for 45 s and last cycle of final extension at 72°C for 5 min. The PCR products containing 5 μl of ethidium bromide were separated by electrophoresis in 2% agarose gel using 1× TAE buffer with Trackit™ 1 kb Plus DNA Ladder (Invitrogen) at 80 V for 40 min.

### 2.8 Sex determination using loop‐mediated isothermal amplification application for field test

A traditional goal of LAMP is to develop noninvasive sampling methods that can be successfully implemented in the field without the use of sophisticated instruments. Hence, we extracted DNA from seventeen feathers using a HotSHOT technique adapted from Truett et al. (2000). 37.5 μl of alkaline lysis reagent were added to the feather sample, and it was submerged as needed. The sample was then heated to 95°C for 20 min. In each tube, 37.5 μl of neutralizer were added, and the sample was mixed by vortexing. The 3 μl of genomic DNA were examined using the proper LAMP reaction and a colorimetric assay. The effectiveness of LAMP reactions was compared using conventional PCR.

## 3 RESULT

### 3.1 Outer primers selection

**FIGURE 4:**
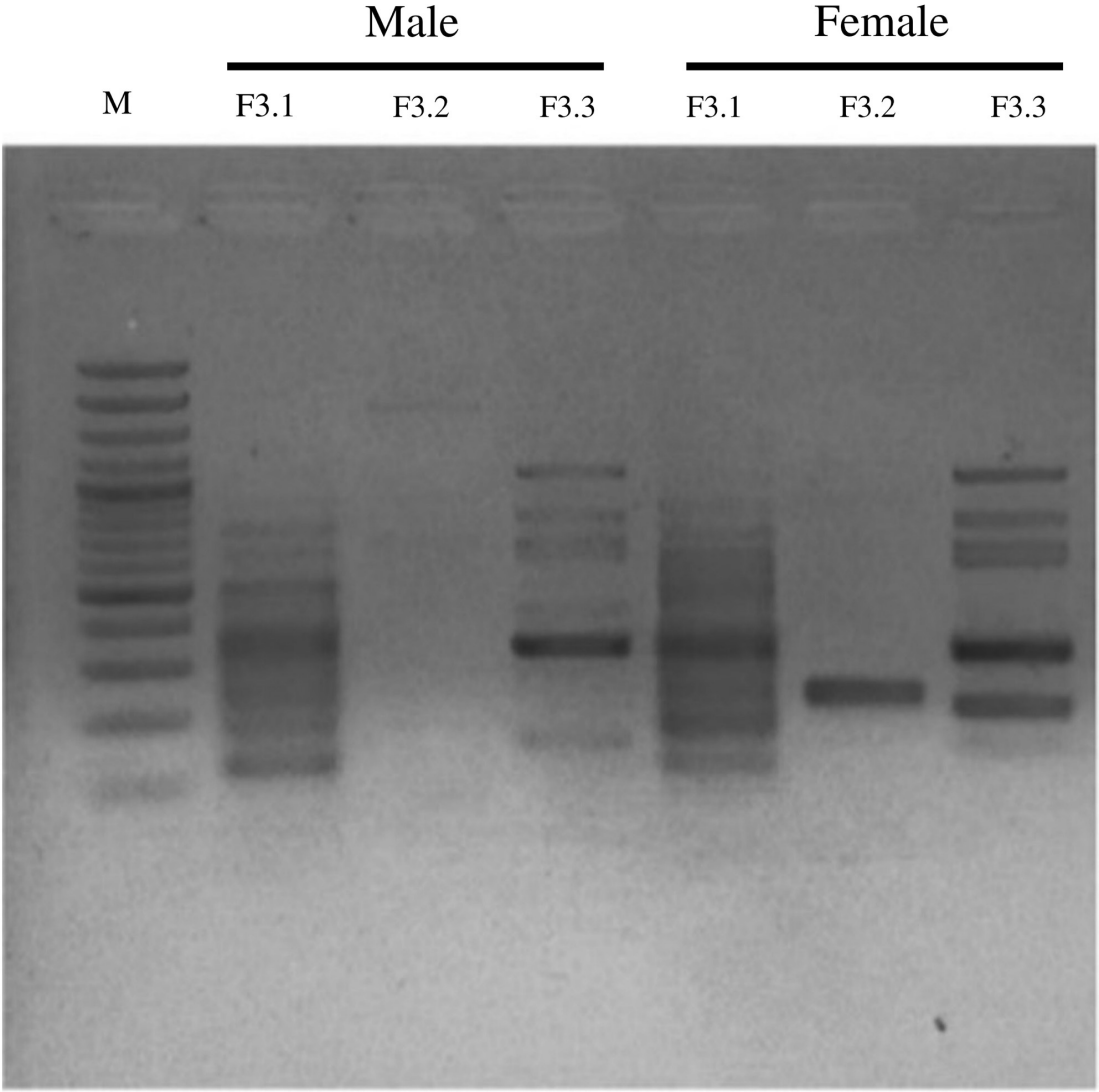
The result of the PCR amplification of *CHD‐W* gene using the candidate outer primer F3.1/B3, F3.2/B3, and F3.3/B3. PCR products are amplified DNA are shown as differences between male and female for F3.2/B3.

**FIGURE 5:**
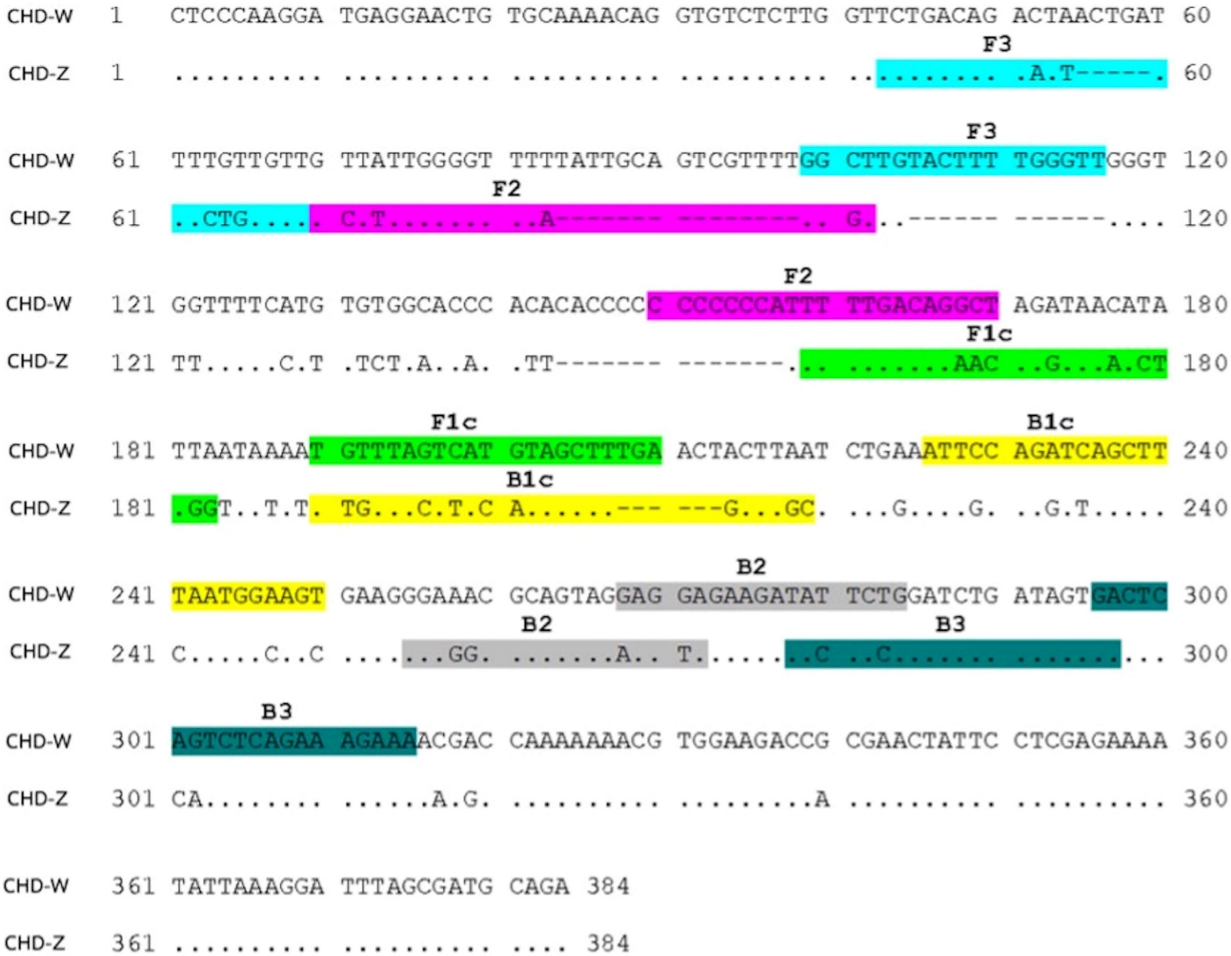
The locations of LAMP primers on the *CHD‐W* and *CHD‐Z* gene of the red‐whiskered bulbul. The upper sequence is the *CHD‐W* gene, while the lower sequence is the *CHD‐Z* gene.

To find the optimal forward outer primer for *CHD‐W* gene amplification of specific product was detected using 1.5% agarose gel electrophoresis. The result showed that the products from primers F3.2/B3 gave amplified fragments that differed significantly between males and females. PCR product from the *CHD‐W* gene is only gave one DNA fragment with approximate size of 220 base pairs in the female, while failed to generate bands in males (Figure 4). The locations of LAMP primers (FIP, BIP, F3, and B3) for the *CHD‐W* and *CHD‐Z* gene amplification is shown in Figure 5.

### 3.2 Optimal condition of loop‐mediated isothermal amplification

For sex identification of red‐whickered bulbuls by LAMP technique, the *CHD* fragments were amplified with two sets of primers: the *CHD‐Z* gene is a control, while the *CHD‐W* gene is used as a gender identifier. The male and female DNA can be amplified with the CHD‐Z primer set, while the CHD‐W primer set only amplifies the female DNA. The optimal combination of the ratio between the outer primers and the inner primers, reaction temperatures, and incubation times showed that CHD‐Z and CHD‐W primers copiously discriminate between females and males when LAMP reactions were performed at 63°C for 80 min and 1:4 ratio of outer primer and inner primer (Figures S1–S8).

### 3.3 Sensitivity of colorimetric assays

**FIGURE 6:**
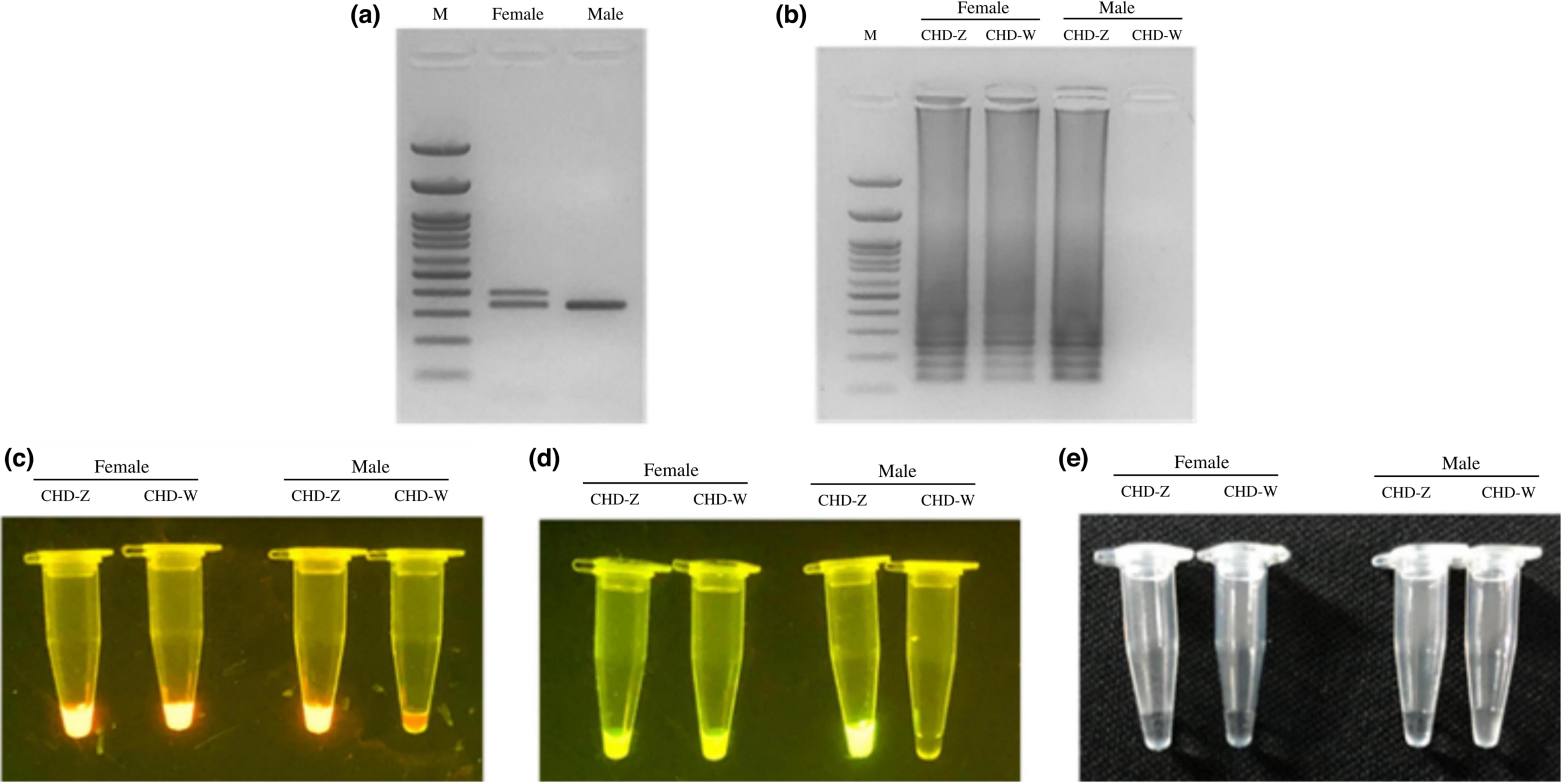
Comparative sex identification of red‐whickered bulbuls between PCR (a) and Post‐LAMP analysis method using electrophoresis (b), colorimetric assay using GelRed® (c), SYTO™ 9 (d), and CuSO_4_ (e).

Electrophoresis and colorimetric assays were used to detect LAMP products. The ladder characteristic of the results can be seen when the red‐whiskered bulbul's DNA was amplified with CHD‐Z primer (all individuals) and CHD‐W primer (only female). Where the amount of DNA was increased in the GelRed® samples, the solutions changed color from transparent to orange and showed a fluorescent glow under UV light. The CHD‐Z and CHD‐W‐specific primers amplified and glowed with the female DNA. However, CHD‐W only reacted with the female DNA and not the male. Corresponding to SYTO™ 9, the solution color changed from clear to green. Female DNA reacted with CHD‐Z and CHD‐W primers and showed a green glow under UV light. Simultaneously, male DNA only reacted with the CHD‐Z primer. As a result, the CHD‐W reaction remained green and did not glow when exposed to UV light. When the product of the LAMP reaction was examined with CuSO_4_, it was found that in female bird samples with increased DNA where the CHD‐Z and CHD‐W primers were used, there was no change in color of the solutions and they remained clear after adding CuSO_4_. When the primer *CHD‐Z* gene was added to male bird samples, the solution with CuSO_4_ showed no change; the solution was clear. The *CHD‐W* gene primer showed more turbid sedimentation. The results of the LAMP reactions were confirmed using the PCR technique, which is the standard method for general bird sex identification. The P2/P8 primer set produced one band of male DNA (334 base pairs), while the DNA of female birds produced two bands of DNA (334 and 384 base pairs; Figure 6).

**FIGURE 7:**
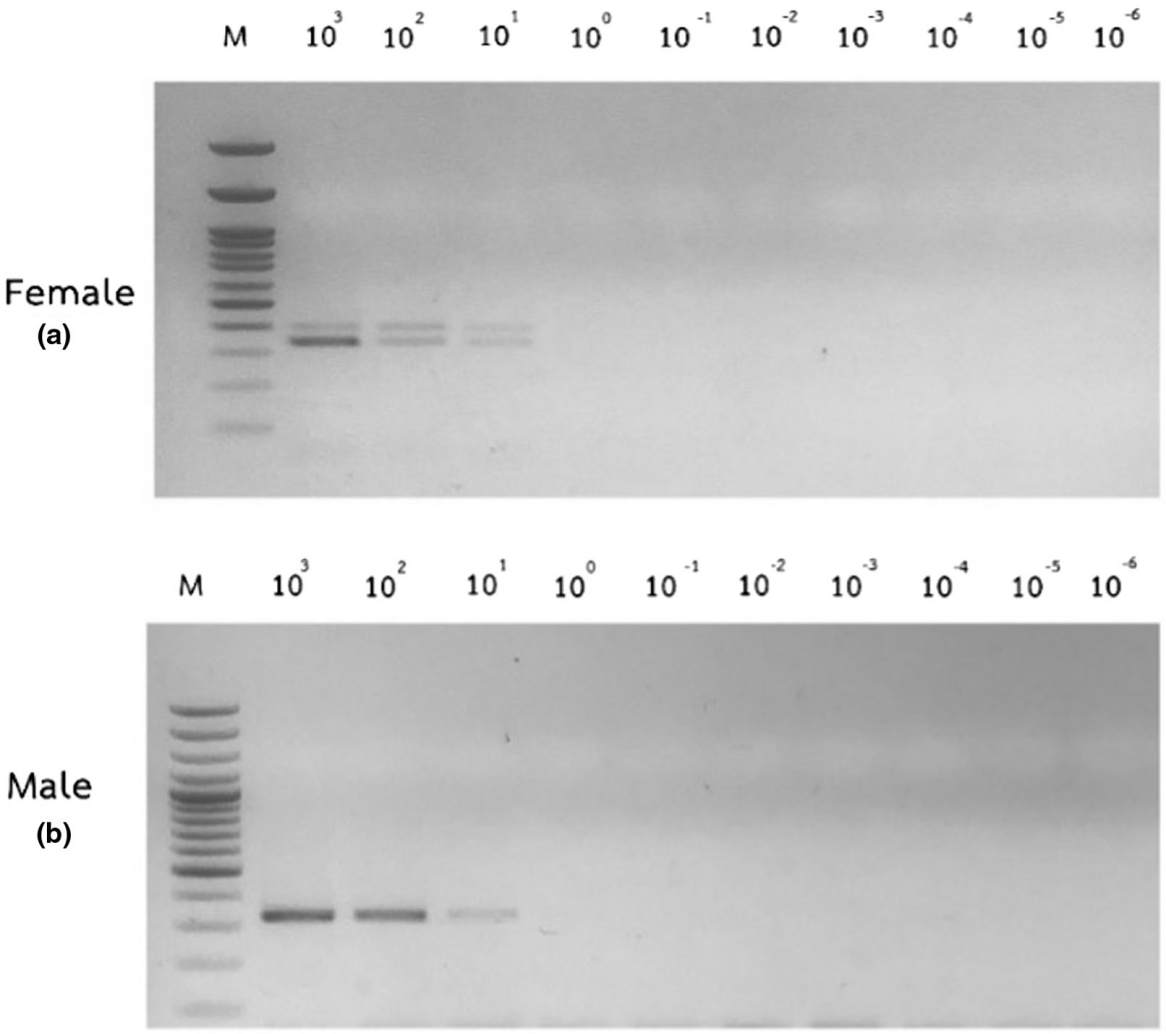
Agarose gel electrophoresis of female (a) and male (b) PCR products generated from primers P2/P8. DNA concentrations were amplified ranging from 10^3^ to 10^−6^ ng.

**FIGURE 8:**
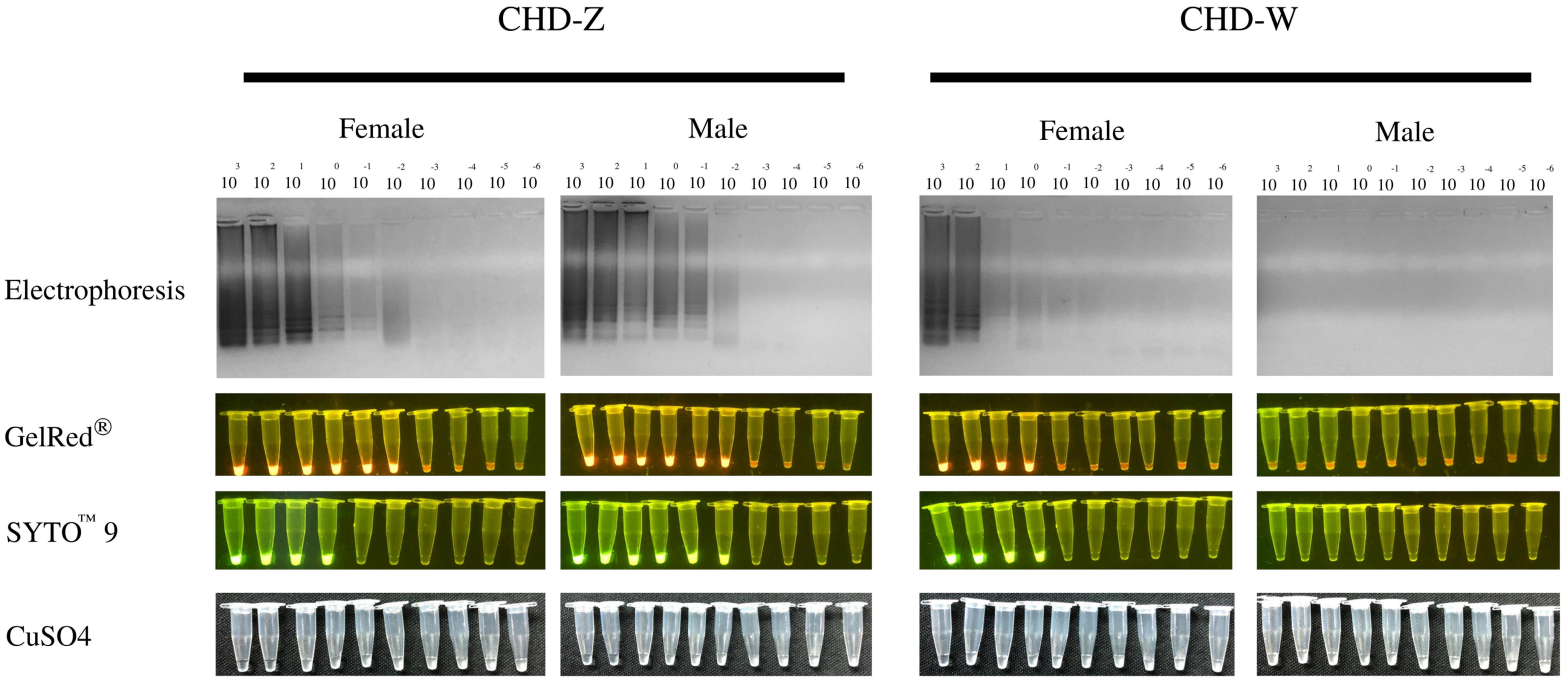
Sensitivity of post‐LAMP analysis method compared with electrophoresis using DNA concentration between 10^3^–10^−6^ ng

Sensitivity of colorimetric assays for LAMP products was compared with PCR technique by conducting amplification using red‐whiskered bulbul's DNA in the range of 10^3^–10^−6^ ng as DNA template. The lowest detectable level of male and female red‐whiskered bulbul DNA by PCR was 10 ng (Figure 7). LAMP product visualization using fluorescent dyes and chemical reactions revealed that GelRed® was the most sensitive assay, followed by SYTO™ 9, and CuSO_4_. The CHD‐Z reaction detected both female and male DNA at 10^−2^ ng. While increasing the DNA template with the CHD‐W reaction, only the LAMP product of female DNA was detected at 1 ng. However, when amplified DNA at 1 ng was stained with GelRed® and SYTO™ 9, the *CHD‐Z* and *CHD‐W* LAMP detection limits were similar and gave accurate results in both sexes (Figure 8).

### 3.4 Specificity of colorimetric assays

The LAMP and Post‐LAMP analysis methods were validated to determine the sex of the red‐whiskered bulbul. The percent accuracy of the post‐LAMP analysis method was calculated from the number of red‐whiskered bulbuls that can correctly identify sex when compared with the results from the PCR technique divided by the total number of red‐whiskered bulbuls in this study, which was 23. The results showed that electrophoresis and the addition of fluorescent dyes, GelRed® and SYTO™ 9, were able to correctly identify the sex of red‐whiskered bulbuls with 100% accuracy. While the CuSO_4_ method, which involved observing the turbid sediment, revealed that only nine of 23 birds were correctly identified as male or female (39.13%; Table 2).

**TABLE 2:** Summary of correct results obtained by LAMP and post‐LAMP analysis method.

No.	Post‐LAMP analysis	Percentage of accuracy
1	Electrophoresis	100% (23/23)
2	GelRed®	100% (23/23)
3	SYTO™ 9	100% (23/23)
4	CuSO_4_	39.13% (9/23)

*Note*: Percentage of accuracy were calculated form bird can sex identify/total number of examined samples.

### 3.5 | Detection of genomic DNA from simple DNA extraction method by loop‐mediated isothermal amplification for field application

A total of 17 feather samples were collected for the LAMP technique combined with GelRed® and SYTO™ 9 for colorimetric assays. The results showed that all samples were positive when tested with the CHD‐Z primer and LAMP products gave orange (GelRed®), or green (SYTO™ 9) in the tested tubes. The results indicated that a simple DNA extraction method (HotSHOT) could be used as a method for providing genomic DNA for LAMP amplification. CHD‐W primer was tested to determine the sex of red‐whiskered bulbuls (Figure S9). According to the findings, when compared to conventional PCR, the genomic DNA obtained through simple DNA extraction was able to totally and accurately distinguish between the sexes of red‐whiskered bulbuls (Table S2).

## 4 DISCUSSION

Nowadays, sex identification through DNA detection is influencing the bird culture industry. DNA amplification from sex chromosomes by PCR has been widely reported, and the specific method has been shown to be highly effective (Griffiths et al., 1998). However, due to time‐consuming protocols and the requirement of specialized laboratory instruments, PCR is not suitable for rapid application and efficient study in field‐scale (Centeno‐Cuadros et al., 2016, 2017; Chan et al., 2012; Koch et al., 2019; Notomi et al., 2000). In this study, sex identification of the red‐whiskered bulbul by the LAMP technique was developed by identifying the *CHD* gene of sex chromosomes Z and W. The CHD‐W reaction distinguishes between males and females because the *CHD‐W* gene is only found in female birds. The CHD‐Z reaction amplified the *CHD‐Z* gene in both male and female samples and served as a control to present sufficient quality and quantity of DNA for detection by the LAMP technique. In addition, the female‐specific sequence and the 18S ribosomal RNA sequence were used for sex identification (Chan et al., 2012). The result indicated that the distances between primers are critical in LAMP for the specificity of LAMP amplification. Particularly, the distance between the 3′ end of the outer primer and the 5′ end of F2 and B2 of the inner primer, in which strand displacement activity appears in this region. The optimal distance between primers is approximately 0–60 base pairs and, as a result, the new DNA can be generated in the loop structure self‐primed strand displacement during LAMP cycling steps allows DNA amplification to begin with an amplified whirl of FIP and BIP primers. In this study, TTTT were used to link between the FIP and BIP primers. However, other nucleotides can be used for links, or without the connection of nucleotides can also amplify the DNA (Chan et al., 2012). Furthermore, the ratio between the outer primer and the inner primer is important for successful DNA amplification in LAMP as well as primer design. The optimal primer ratio necessitates working with *Bst* DNA polymerase to generate dumbbell‐shaped DNA structures. In the cycle amplification step, the concentration of the inner primer had an effect on the LAMP reaction. The inner primer would work in conjunction with the loop region of the dumbbell‐shaped DNA structures. When it becomes interconnected in the loop region, a reaction occurs, requiring extensive resources to build new DNA. LAMP necessitates such resources because it generates a large amount of DNA. In the study conducted by Chan et al. (2012), the optimal ratio of outer primer to inner primer was found to be between 1:4 and 1:12 (Chan et al., 2012). According to the findings of this study, a 1:4 ratio may rapidly increase the ability to amplify DNA in both reactions while providing consistent results.

The temperatures and incubation times were optimized and found that incubating at 63°C for 80 min was the best condition for DNA amplification using LAMP technique for determining the sex of red‐whiskered Bulbul. This condition is clearly determined for ladder pattern characteristics in the *CHD‐Z* and *CHD‐W* genes. The ideal temperature for LAMP is determined by enzyme activity. In the amplifying DNA step, the *Bst* DNA polymerase enzyme performs best at 60–65°C (Dhama et al., 2014; Notomi et al., 2000) Various methods for visualizing LAMP products without gel electrophoresis have been reported (Moonga et al., 2020; Papadakis et al., 2022; Poole et al., 2017; Sharma et al., 2021; Wong et al., 2017). In this experiment, adding GelRed®, SYTO™ 9, and CuSO_4_ were selected to find the optimal method for checking LAMP products from CHD‐Z and CHD‐W reactions. GelRed® is a fluorophore, it has optical properties similar to ethidium bromide. It fluoresces an orange color when exposed to ultraviolet light, which intensifies after binding to DNA. The compound is promoted as a safer and more sensitive alternative to ethidium bromide. The results showed that using GelRed® and SYTO™ 9 could check the products at the level of DNA, with greater sensitivity than CuSO_4_ because it is a direct product examination matches with the increased double helix DNA (Figure 8; Hao et al., 2018; Subharat et al., 2011). Characteristically, GelRed® and SYTO™ 9 are fluorescent substances that capture single‐strand and double‐strand DNA (Horáková et al., 2011). As a consequence, nonspecific DNA that is not the detection target can be captured (Hardinge & Murray, 2019). Therefore, precise DNA amplification is required to monitor LAMP reaction yields. GelRed® had been reported to examine the LAMP product of goose circovirus detection. The result showed consistency of LAMP product when analyzed by electrophoresis and GelRed®. The LAMP assay can examine the samples when added GelRed® after 30 min (Woźniakowski et al., 2012). In the case of SYTO™ 9, it was used to detect the products in the real‐time LAMP. Real‐time system measurement demonstrated that the signal of SYTO™ 9 was the highest when compared to other SYTO‐types. The lowest initial DNA concentration that could be examined was 2 pg. The increased amount of DNA from the LAMP technique can give a greater amount of fluorescent light. Increasing SYTO™ 9 concentration was found to reduce the time for DNA testing. The suitable concentration of SYTO™ 9 is 2–10 μM (Quyen et al., 2019). Unlike GelRed® and SYTO™ 9, CuSO_4_ measures DNA indirectly by binding the dNTPs that remain after the LAMP reaction (Kanchanaphum, 2018). When CuSO_4_ was added to the negative sample, a white ring of Cu(OH)_2_ precipitated. The ring could not be seen clearly in this study, and turbidity appeared in the solution. The reason could be due to the small volume of the LAMP reaction. A report stated that 100 mM CuSO_4_ was used to test the product of the LAMP reaction in human DNA (Kanchanaphum, 2018) and cattle sex identification (Boonkong et al., 2013b). However, the result of checking the LAMP product with CuSO_4_ showed lower accuracy (Boonkong et al., 2013a).

In this study, it was discovered that when LAMP products were electrophoretically analyzed, the results from the LAMP reaction using the primer for the *CHD‐Z* gene showed smearing bands by increasing amounts of both male and female DNA at 10^−2^ ng. Smearing bands were observed for the CHD‐W primer as female DNA concentration was increased, starting at 10 ng. At 1 ng, the DNA concentration was found to be smearing but not very clear. For the method of checking the LAMP product by colorimetric assays, it was found that GelRed® can verify the product from the LAMP reaction that was increased in amount by the CHD‐Z primer set from the initial concentration of DNA at 10^−2^ ng. In male and female samples, which corresponds to the electrophoresis examination of the results. While the CHD‐W primer was able to examine the product of female DNA at the DNA level starting at 1 ng, which is noticeably easier than the electrophoresis method, product examination by electrophoresis at the DNA concentration as low as 1 ng is difficult to observe the smearing characteristics. For examination of the LAMP product by colorimetric assays method using SYTO™ 9 found that it was able to check the product from CHD‐Z primers in increasing the amount of female DNA in concentration at 1 ng. While male DNA can check the product in initial concentration at 10^−2^ ng, which is more sensitive than females. It could be due to the fact that male DNA has two Z chromosomes while female DNA only has one. The CHD‐W primer set can check the product of female DNA by increasing the amount of DNA. The initial concentration at 1 ng, which is the DNA concentration that is easier to detect when compared with the electrophoresis method, which has the same result as when using GelRed®. After testing the LAMP product with CuSO_4_, the results revealed that the CHD‐Z primer set could be used with higher concentrations of male and female DNA, starting at 10^2^ ng. While higher the amount of DNA with the CHD‐W primer set can check the output from females at a concentration of 10^3^ ng. It was found to be less sensitive than the electrophoresis method because product examination by the CuSO_4_ method is complicated because there is only a minor difference in reaction tubes or nonreaction tubes due to the reaction appearing turbid or not turbid, which can be difficult to see. The reaction is clearly visible as a glow in other methods.

When compared to the PCR standard method, the LAMP technique was found to correctly distinguish the sex of the red‐whiskered bulbul 100% of the time using P2 and P8 primers (Changtor & Yimtragool, 2020; Tana & Panprommin, 2014). LAMP identified 14 males and nine females. The electrophoresis results of the LAMP technique showed that the DNA band formed with Ladder‐like characteristics and that the results were 100% accurate. The DNA band that formed was visible and distinct. The LAMP product was tested using a colorimetric assay that included GelRed®, SYTO™ 9, and CuSO_4_. The results showed that the GelRed® and SYTO™ 9 methods for examining LAMP products are as accurate as the conventional PCR method. It can provide 100% accuracy because GelRed® and SYTO™ 9 have the ability to directly check the amount of DNA by binding in a double‐strand DNA structure. While LAMP product assay with CuSO_4_ showed 39% accuracy of LAMP detection. However, it is difficult to observe the ring formed and turbidity of LAMP reaction. Corresponding to the report of Boonkong et al. (2013a), the results showed low accuracy of using CuSO_4_ to examine LAMP reaction for sexual segregation of ruminant embryos (35% accuracy in bovine and 50% accuracy in goat; Boonkong et al., 2013b). The low concentration of DNA templates may affect the chemical reaction between CuSO_4_ and dNTPs. DNA templates for validation in this experiment were extracted from blood spots using lysis buffer, which is the easy method and uses a low volume of blood samples. The concentration of DNA extracted by lysis buffer ranged from 72.4 to 422.4 ng/μl (Table S1). As a result, the sensitivity of CuSO_4_ cannot detect LAMP products from small amounts of DNA. When the time for sex identification was compared between the LAMP method analyzed with GelRed® and SYTO™ 9 and PCR with gel electrophoresis, the LAMP method took only 80 min, while the PCR with gel electrophoresis took more than 3 h. As a result, the LAMP method and GelRed® and SYTO™ 9 analysis are suitable methods for red‐whiskered Bulbul sex identification.

In essence, the LAMP methodology was developed for field research, and this study has extended our understanding of the factors influencing performance and field matters pertaining to DNA extraction methods. Therefore, it was necessary to simplify DNA extraction for the field test. The successful use of feathers as a source of DNA can decrease time for sex identification when combined with a simple DNA extraction method. The result indicated that the feathers can be used as noninvasive samples for bird sex identification in field studies when combined with the LAMP technique. In this study, DNA extracted using the HotSHOT technique was amplified using the LAMP technique with the CHD‐Z primer and yielded 100% success in all tested tubes. The findings indicated that DNA extracted from feathers using the HotSHOT technique could be a reliable source of genomic DNA. Moreover, the HotSHOT solution's components are simple to prepare: NaOH and disodium EDTA are used as lysis reagents, and Tris–HCl is used to neutralize the solution (Truett et al., 2000). In reviewing the literature, DNA from the HotSHOT is extensively sheared and at a low concentration, making it unsuitable for genotyping studies (Truett et al., 2000). However, the LAMP technique can amplify HotSHOT DNA without false negatives. The LAMP technique is very sensitive and can successfully utilize DNA obtained from HotSHOT. When using CHD‐W primer, DNA extracted using HotSHOT technique has a sex identification success rate of up to 100% (Table S2). The outcome demonstrated that the HotSHOT technique and the LAMP technique could be combined for the onsite sex determination. Sex determination in the field is important for population structure management; conservation efforts depend on the sex ratio being balanced in small populations (Cerit & Avanus, 2007). As a result, data on population size and sex ratio are required for effective conservation management (Chang et al., 2012), and sex identification using the LAMP technique in a field test can be an impact tool for research.

## 5 CONCLUSIONS

This study showed our successful development of the CHD‐Z and CHD‐W primer sets of the LAMP method for the molecular identification of the red‐whiskered bulbul. This method can be completed quickly and without the use of sophisticated equipment. The sensitivity of the LAMP technique and some colorimetric assays, namely, GelRed® and SYTO™ 9, when combined to examine the post‐LAMP analysis, is higher than conventional PCR‐based methods for sex determination. LAMP colorimetric assays have the same specificity as PCR and an accuracy of up to 100%. In the future, ecologists, bird breeders, and commercial farmers may use this application to replace conventional PCR‐based sex determination methods.

## AUTHOR CONTRIBUTIONS

**Phanupong Changtor:** Conceptualization (equal); data curation (equal); formal analysis (equal); investigation (equal); methodology (lead); visualization (lead); writing – original draft (lead). **Yash Munnalal Gupta:** Conceptualization (equal); investigation (equal); methodology (supporting); visualization (supporting); writing – review and editing (supporting). **Nonglak Yimtragool:** Conceptualization (equal); data curation (equal); formal analysis (equal); funding acquisition (lead); investigation (equal); methodology (equal); project administration (lead); visualization (equal); writing – original draft (equal); writing – review and editing (lead).

## CONFLICT OF INTEREST

The authors declare no conflict of interest.

## FUNDING INFORMATION

This work was supported by Faculty of Science, Naresuan University (grant number is R2565E028) about informaion of funding appear in last sentence.

## Supporting information


Appendix S1

## Data Availability

No data were produced.
