# Supplementary material for: Optimization and application of loop‐mediated isothermal amplification technique for sex identification in red‐whiskered bulbul (Pycnonotus jocosus)
Source: Ecol Evol. 2022 Oct 5;12(10):e9401. doi: 10.1002/ece3.9401 (PMC9534725; doi:10.1002/ece3.9401)
Supplement: Supplementary file 1 — Appendix S1 [file ECE3-12-e9401-s001.docx]

**Supplemental Information for:**

**Optimization and Application of LAMP Technique for Sex Identification in Red‐Whiskered Bulbul (*Pycnonotus jocosus*)**

**Phanupong Changtor, Yash Munnalal Gupta and Nonglak Yimtragool**

**Table of Contents:**

| **Figure S1** | **Page 1** |
| --- | --- |
| **Figure S2** | **Page 2** |
| **Figure S3** | **Page 3** |
| **Figure S4** | **Page 4** |
| **Figure S5** | **Page 5** |
| **Figure S6** | **Page 6** |
| **Figure S7** | **Page 7** |
| **Figure S**8 | **Page** 8 |
| **Figure S**9 | **Page** 9 |
| Table S1 | **Page** 10 |
| Table S2 | **Page** 11 |


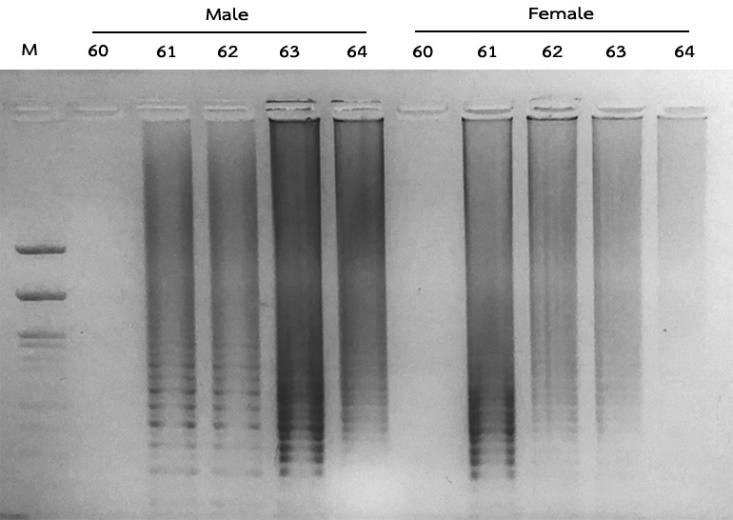


**Figure S1.** The result of the LAMP amplification of *CHD-Z* gene using CHD-Z primer. Lane 1-5 are male samples at 60-64 °C, and Lane 6-10 are female samples at 60-64 °C.


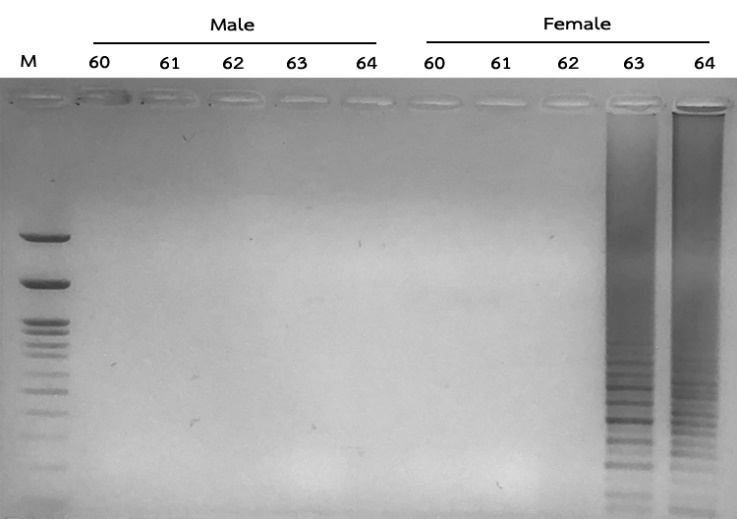


**Figure S2.** The result of the LAMP amplification of *CHD-W* gene using CHD-W primer. Lane 1-5 are male samples at 60-64 °C, and Lane 6-10 are female samples at 60-64 °C.


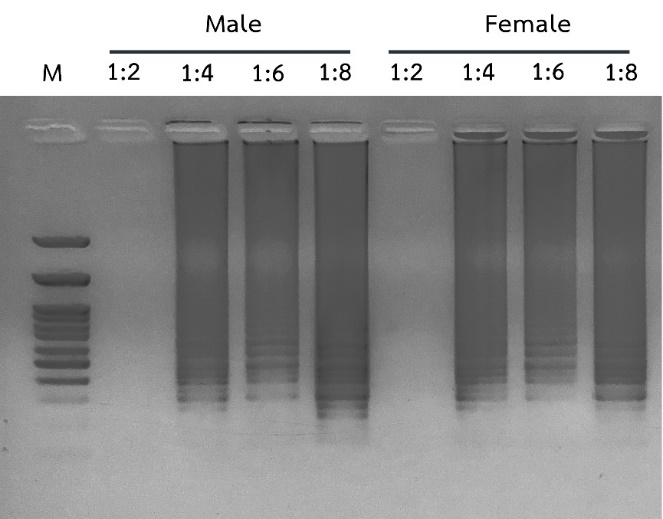


**Figure S3.** The result of the LAMP amplification of *CHD-Z* gene using CHD-Z primer. Lane 1-4 are male samples using ratio of outer primer : inner primer at 1:2, 1:4, 1:6 and 1:8. Lane 5-8 are female samples using ratio of outer primer : inner primer at 1:2, 1:4, 1:6 and 1:8


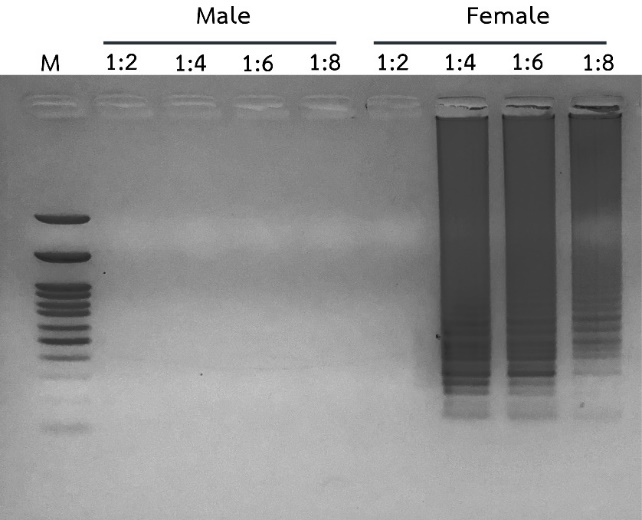


**Figure S4.** The result of the LAMP amplification of *CHD-W* gene using CHD-W primer. Lane 1-4 are male samples using ratio of outer primer : inner primer at 1:2, 1:4, 1:6 and 1:8. Lane 5-8 are female samples using ratio of outer primer : inner primer at 1:2, 1:4, 1:6 and 1:8


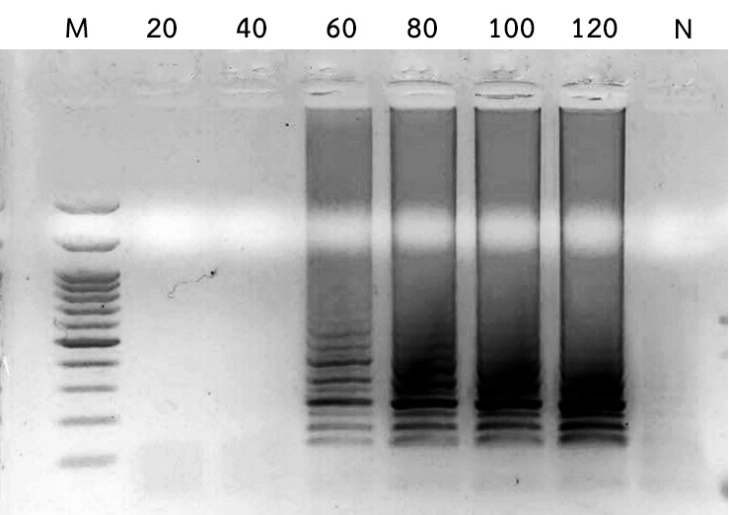


**Figure S5.** The effect of incubation times of the female sample using LAMP amplification with CHD-Z primer. Lane 1-6 are 20, 40, 60, 80, 100, and 120 minute, respectively.


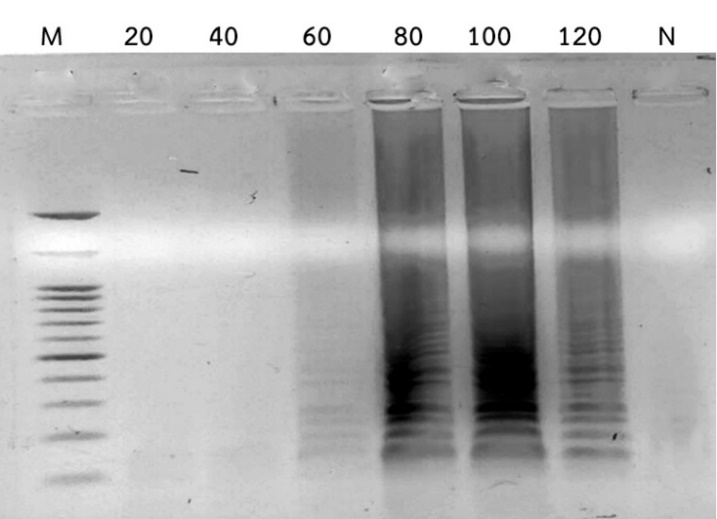


**Figure S6.** The effect of incubation times of the female sample using LAMP amplification with CHD-W primer. Lane 1-6 are 20, 40, 60, 80, 100, and 120 minute, respectively.


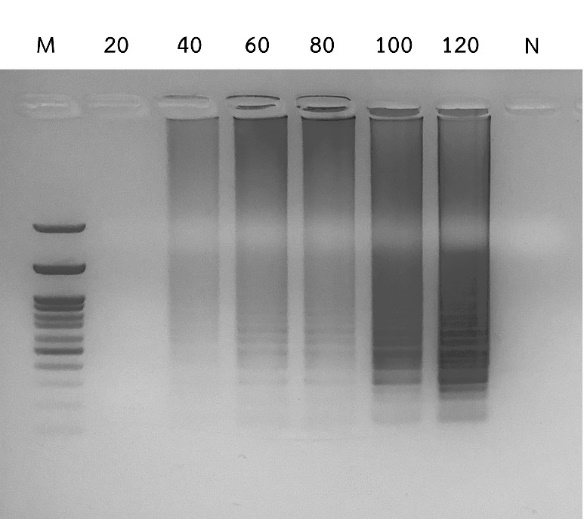


**Figure S7.** The effect of incubation times of the male sample using LAMP amplification with CHD-Z primer. Lane 1-6 are 20, 40, 60, 80, 100, and 120 minute, respectively.


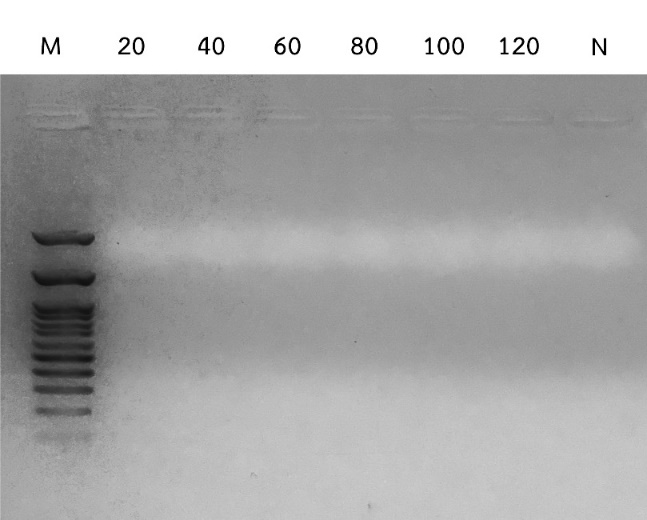


**Figure S8.** The effect of incubation times of the male sample using LAMP amplification with CHD-W primer. Lane 1-6 are 20, 40, 60, 80, 100, and 120 minute, respectively.

**Figure S9.** Sex identification of red-whickered bulbuls by LAMP combined with GelRed^®^ and SYTO™ 9 from the feather sample using simple DNA extraction method (HotSHOT).

**Table 1S.** DNA concentration and purity from the blood sample obtained with lysis buffer.

| Number | Sex. | DNA concentration | Purity |
| --- | --- | --- | --- |
|  |  | (ng/μl) | (A_260_/A_280_) |
| 1 | Male | 260.6 | 1.39 |
| 2 | Female | 250.6 | 1.4 |
| 3 | Male | 195.4 | 1.32 |
| 4 | Male | 173.2 | 1.3 |
| 5 | Male | 72.4 | 1.19 |
| 6 | Female | 207.7 | 1.32 |
| 7 | Female | 155.4 | 1.44 |
| 8 | Female | 263 | 1.41 |
| 9 | Male | 422.4 | 1.39 |
| 10 | Male | 239.4 | 1.4 |
| 11 | Female | 374.6 | 1.48 |
| 12 | Male | 343.5 | 1.43 |
| 13 | Female | 184.7 | 1.46 |
| 14 | Male | 164.3 | 1.44 |
| 15 | Male | 122.8 | 1.46 |
| 16 | Female | 214.2 | 1.45 |
| 17 | Male | 166.9 | 1.46 |
| 18 | Male | 123.3 | 1.47 |
| 19 | Female | 171 | 1.56 |
| 20 | Male | 357.4 | 1.44 |
| 21 | Female | 128.9 | 1.53 |
| 22 | Male | 132.8 | 1.49 |
| 23 | Male | 271 | 1.6 |

**Table 2S.** DNA concentration, purity and result of sex identification from the feather sample using simple DNA extraction method (HotSHOT).

| Number | Sex | DNA concentration | Purity | Result of sex identification. |
| --- | --- | --- | --- | --- |
|  |  | (ng/μl) | (A_260_/A_280_) |  |
| 1 | Male | 51.2 | 1.36 | √ |
| 2 | Male | 37.7 | 0.84 | √ |
| 3 | Male | 51.0 | 1.26 | √ |
| 4 | Female | 32.7 | 0.96 | √ |
| 5 | Female | 49.1 | 1.15 | √ |
| 6 | Male | 38.0 | 1.01 | √ |
| 7 | Male | 53.2 | 1.14 | √ |
| 8 | Male | 35.2 | 1.03 | √ |
| 9 | Female | 40.2 | 1.09 | √ |
| 10 | Male | 159.5 | 1.24 | √ |
| 11 | Male | 29.2 | 1.04 | √ |
| 12 | Male | 19.2 | 0.99 | √ |
| 13 | Male | 112.9 | 1.26 | √ |
| 14 | Female | 115.9 | 1.44 | √ |
| 15 | Female | 40.5 | 1.29 | √ |
| 16 | Male | 113.4 | 1.24 | √ |
| 17 | Female | 27*.*3 | 0.95 | √ |
